# Evaluation of Anti-VEGFR2 Specific Photoimmunotherapy for Targeted Regression of Neovascularization in an AMD Model

**DOI:** 10.1167/iovs.66.6.70

**Published:** 2025-06-23

**Authors:** Hideto Osada, Takashi Nishimura, Makoto Mitsunaga, Masayuki Saruta, Kazuo Tsubota, Kazuno Negishi, Toshihide Kurihara, Norimitsu Ban

**Affiliations:** 1 Laboratory of Aging and Retinal Biology, Keio University School of Medicine, Tokyo, Japan; 2 Department of Ophthalmology, Keio University School of Medicine, Tokyo, Japan; 3 Division of Gastroenterology and Hepatology, Department of Internal Medicine, The Jikei University School of Medicine, Tokyo, Japan; 4 Tsubota Laboratory, Inc., Tokyo, Japan; 5 Laboratory of Photobiology, Keio University School of Medicine, Tokyo, Japan

**Keywords:** age-related macular degeneration, photoimmunotherapy

## Abstract

**Purpose:**

This study aimed to evaluate the efficacy of photoimmunotherapy (PIT) targeting VEGFR2 for the treatment of neovascular AMD and to investigate its potential as a novel therapeutic strategy.

**Methods:**

DC101-IR700, a conjugate of the anti-mouse VEGFR2 monoclonal antibody DC101 and the photosensitizer IR700, was investigated both in vitro and in vivo. VEGFR2 expression in endothelial cells was confirmed via qPCR and immunocytochemistry. Laser-induced choroidal neovascularization (CNV) was established in C57BL/6J mice. Localization of DC101-IR700 within CNV lesions was assessed by immunofluorescence. After PIT was performed using either a 690 nm near-infrared manual laser or a slit lamp laser, CNV volumes were quantified through confocal microscopy. Cell viability post PIT was measured using MTT assay and cell death in CNV lesions was evaluated using TUNEL staining.

**Results:**

DC101-IR700 localized specifically to VEGFR2-positive cells in CNV lesions, and PIT induced significant VEGFR2-dependent cytotoxicity in vitro. In vivo, both PIT and directional PIT using slit lamp laser significantly reduced CNV volumes compared with controls. TUNEL staining confirmed VEGFR2-specific cell death in treated CNV lesions. Directional PIT achieved similar efficacy to PIT, demonstrating its potential as a clinically viable alternative.

**Conclusions:**

PIT targeting VEGFR2 selectively induced cell death in pathological neovascular tissues, significantly reducing CNV volume in an AMD model. These findings suggest that VEGFR2-specific PIT represents a promising and targeted approach for treating neovascular AMD, offering advantages over conventional anti-VEGF therapies by potentially decreasing treatment frequency and improving efficacy.

Abnormal neovascularization is a pathological feature of various ocular diseases that lead to significant vision loss, such as AMD, diabetic retinopathy, and retinopathy of prematurity.^1^^–^^3^ Neovascular AMD, also known as wet AMD, is a cause of age-related visual impairment and is an important disease that causes vision loss especially in the elderly.^4^^,^^5^ Neovascular AMD is characterized by abnormal choroidal neovascularization (CNV) that causes fluid to leak beneath the retina.^2^ VEGF promotes the proliferation, migration, and survival of endothelial cells, contributing to the development of new, often leaky, blood vessels that compromise the structural and functional integrity of the retina.^6^ Although large studies have demonstrated the benefits of regular treatments with VEGF inhibitors,^7^^,^^8^ leading to anti-VEGF therapies as the standard treatment for neovascular AMD, they are associated with several limitations, including the need for frequent intravitreal injections, incomplete response in some patients,^9^^–^^11^ and potential systemic and ocular adverse effects owing to the broad inhibition of VEGF signaling.^12^

Given these limitations, there is a growing interest in developing more targeted therapies that can disrupt pathological angiogenesis specifically without compromising normal physiological processes.^13^ One such approach is the use of monoclonal antibodies that target VEGF receptor 2 (VEGFR2), a key receptor in VEGF signaling that is expressed predominantly on vascular endothelial cells involved in angiogenesis.^14^^,^^15^ By inhibiting VEGFR2 activation, anti-VEGFR2 antibody could prevent the downstream signaling responsible for angiogenesis.^14^ Previous studies have demonstrated the efficacy of anti-VEGFR2 antibody in preclinical models of cancer and ocular neovascularization, highlighting its potential as a therapeutic agent for conditions characterized by pathological angiogenesis.^16^^–^^18^ VEGFR2-targeted therapies have also shown therapeutic benefits in experimental AMD models,^19^ although it has not yet been applied in clinical practice.

In recent years, the concept of photoimmunotherapy (PIT) has emerged as a novel strategy for cancer therapy that combines the specificity of monoclonal antibodies with the light-activated photosensitizer IR700 (IRDye700DX).^20^ Upon exposure to near-infrared (NIR) light, the photosensitizer is activated, inducing rapid and targeted cell-specific necrosis.^21^ A recent clinical trial using the anti–epidermal growth factor receptor antibody cetuximab in PIT for head and neck cancer has advanced to a phase 3 clinical trial (NCT03769506), demonstrating its promise in treating solid tumors. Furthermore, previous studies have demonstrated the anti-carcinogenic effect of PIT using trastuzumab conjugated to IR700 (Tra-IR700) for human epidermal growth factor receptor 2–positive cancer.^22^ Notably, the anticarcinogenic effects of DC101-IR700, a conjugate of the anti-mouse VEGFR2 monoclonal antibody DC101 and IR700, targeting neovascularization in xenograft tumors for gastric cancer have been demonstrated and PIT-induced regression of microvessels has been observed.^23^

This study aimed to evaluate whether DC101-IR700–mediated PIT can be used as an effective treatment for neovascular AMD by conducting animal experiments. Specifically, we sought to assess the in vivo and in vitro localization of DC101-IR700 in models of CNV and to investigate the therapeutic efficacy of DC101-IR700-mediated PIT in reducing CNV volumes in an AMD model. By focusing on the binding specificity and therapeutic potential of DC101-IR700, this research seeks to advance the development of more targeted and effective treatments for neovascular AMD.

## Methods

### Reagents

DC101 was purchased from BioXcell (West Lebanon, NH, USA). IRDye700DX N-hydroxysuccinimide ester (IR700) was obtained from LICOR Biosciences (Lincoln, NE, USA) and Alexa Fluor488 NHS ester (Alexa488) from Life Technologies (Gaithersburg, MD, USA). DC101-IR700 and DC101-Alexa488 were synthesized as previously reported.^23^

### Animals

All animal experiments were performed in accordance with the National Institutes of Health guidelines for work with laboratory animals and the ARVO Animal Statement for the Use of Animals in Ophthalmic and Vision Research after approval by the Institutional Animal Care and Use Committee at Keio University (Approval number; A2021-056). C57BL6/J mice were purchased from CLEA Japan (Tokyo, Japan) and were maintained in standard transparent mouse cages in an air-conditioned room at 23 ± 3°C under a 12-hour dark/light cycle with ad libitum access to food and water.

### Laser-induced CNV

The mice's pupils were dilated with 0.5% tropicamide and 0.5% phenylephrine eyedrops (Santen Pharmaceutical, Osaka, Japan) and anesthetized with intraperitoneal injection of combined anesthetics (midazolam 4 mg/kg body weight [BW] [Sandoz, Tokyo, Japan], medetomidine 0.75 mg/kg BW [Nippon Zenyaku Kogyo, Fukushima, Japan], and butorphanol tartrate 5 mg/kg BW [Meiji Seika Pharma, Tokyo, Japan]). The laser procedure was performed as previously reported.^24^ A 532-nm argon laser (200 mW, 100 ms, 75 mm) was used to irradiate each eye at four spots between retinal vessels and two disc diameters from the optic nerve head. During laser irradiation, bubbles were used as an indicator of Bruch's membrane destruction. Hemorrhaging laser lesions were excluded from data analysis.

### PIT

Four days after CNV induction, 100 µg of DC101-IR700 was administered intraperitoneally. After 24 hours, the mice underwent to laser irradiation after pupil dilation and anesthesia. For PIT, a 690-nm continuous wave manual laser MLL-III-690 (CNI Laser, China) was used to irradiate the entire fundus. The power density was measured with an optical power meter PM100 (Thorlabs, Newtson, NJ, USA). In the case of directional PIT, the slit lamp laser VISULAS 690S (Zeiss, Jena, Germany) was applied to the lesion areas under direct observation through a slit lamp microscope.

### CNV Volume Measurement

Five days after PIT, mice were sacrificed by cervical dislocation under anesthesia and the eyes were enucleated and fixed with 4% PFA for 1 hour. The choroid–sclera complex was isolated and post-fixed in 4% PFA overnight at 4°C. After washing, the tissue was permeabilized with 0.5% Triton X-100 and blocked with 3% BSA and stained with FITC conjugated isolectin B4 (1:500, Vector Laboratories, Burlingame, CA, USA) overnight at 4°C. CNV was observed with the FV4000 confocal laser scanning microscope (Olympus, Tokyo, Japan). Three-dimensional images of CNV were generated and the volume was measured using Imaris (Oxford Instrument, Abingdon, UK).

### Cell Culture

The mouse cell line, bEnd.3 (American Type Culture Collection, Manassas, VA, USA) was maintained in Dulbecco's modified Eagle's medium (DMEM) (Nacalai Tesque, Kyoto, Japan) supplemented with 10% fetal bovine serum (Life Technologies), 100 U/mL penicillin, and 100 µg/mL streptomycin (Fujifilm Wako Pure Chemical, Osaka, Japan).

### Immunofluorescence Staining of DC101-IR700 In Vitro

Cells were seeded on cell culture dishes and incubated for 24 hours at 37°C. The medium was replaced with fresh DMEM containing DC101-IR700 (10 µg/mL). Cells were incubated for another 24 hours at 37°C, washed with PBS, and fixed in 4% PFA for 1 hour. Cells were incubated with anti-VEGFR2 antibody (1:1000, Abcam, Cambridge, UK). Signals were detected using Alexa488-conjugated goat anti-rabbit IgG and Alexa555-conjugated goat anti-rat IgG, respectively.

### In Vitro PIT and Cytotoxicity Assay

After 24 hours incubation with DC101-IR700 (10 µg/mL) at 37°C, cells were washed with PBS and fresh phenol red-free DMEM was added. Cells were irradiated with either manual laser or slit lamp laser. At 24 hours after irradiation, cytotoxicity assay was performed using Cell Proliferation Kit I (Roche, Basel, Switzerland) according to the manufacturer's protocol. Absorbance at 580nm was measured using plate reader Cytation 5 (Agilent Technologies, Santa Clara, CA, USA).

### Real-Time PCR

Total RNA was isolated with TRI REAGENT (Molecular Research Center, Cincinnati, OH, USA) and reverse transcribed using ReverTra Ace qPCR RT kit (Toyobo, Tokyo, Japan). Real-time PCR was performed using the StepOnePlus PCR system (Applied Biosystems, Waltham, MA, USA) and gene expression was quantified using the 2-ΔΔCT method and normalized to GAPDH.

### Immunofluorescence Imaging of Choroidal Flatmounts

At 24 hours after injection of DC101-Alexa488, mice were sacrificed by cervical dislocation under anesthesia and the eyes were enucleated and fixed with 4% PFA for 1 hour. The choroid–sclera complex was isolated and post-fixed in 4% PFA overnight at 4°C. After washing, the tissue was permeabilized with 0.5% Triton X-100 and blocked with 3% BSA, then incubated with anti-VEGFR2 antibody overnight at 4°C. Signals were detected using Alexa546-conjugated goat anti-rabbit IgG. Alexa488 fluorescence from the injected DC101-Alexa488 was visualized directly without further staining.

### Histological Analyses

At 24 hours after DC101-Alexa488 injection, enucleated eyes were embedded in Optimal Cutting Temperature compound (Sakura Finetek Japan, Tokyo, Japan), rapidly frozen using liquid nitrogen, and sectioned at 7 µm. For immunostaining, cryosections were incubated with anti-VEGFR2 antibody and signals were detected using Alexa555-conjugated goat anti-rabbit IgG and Alexa647-conjugated goat anti-rat IgG, respectively. For TUNEL staining, the Click-iT Plus TUNEL Assay Kits for In Situ Apoptosis Detection (ThermoFisher Scientific, Waltham, MA, USA) was used according to the manufacturer's protocols.

### Statistical Analysis

Data were shown as means ± SDs. Statistical analyses were performed using one-way ANOVA with Tukey's post hoc tests for comparisons among three or more groups or, for comparisons between two groups, two-tailed Student's *t* tests or Fisher's exact tests using SPSS Statistics 27 (IBM, Armonk, NY, USA).

## Results

### In Vitro Efficacy of PIT in VEGFR2-Expressing Cells

**Figure 1.**
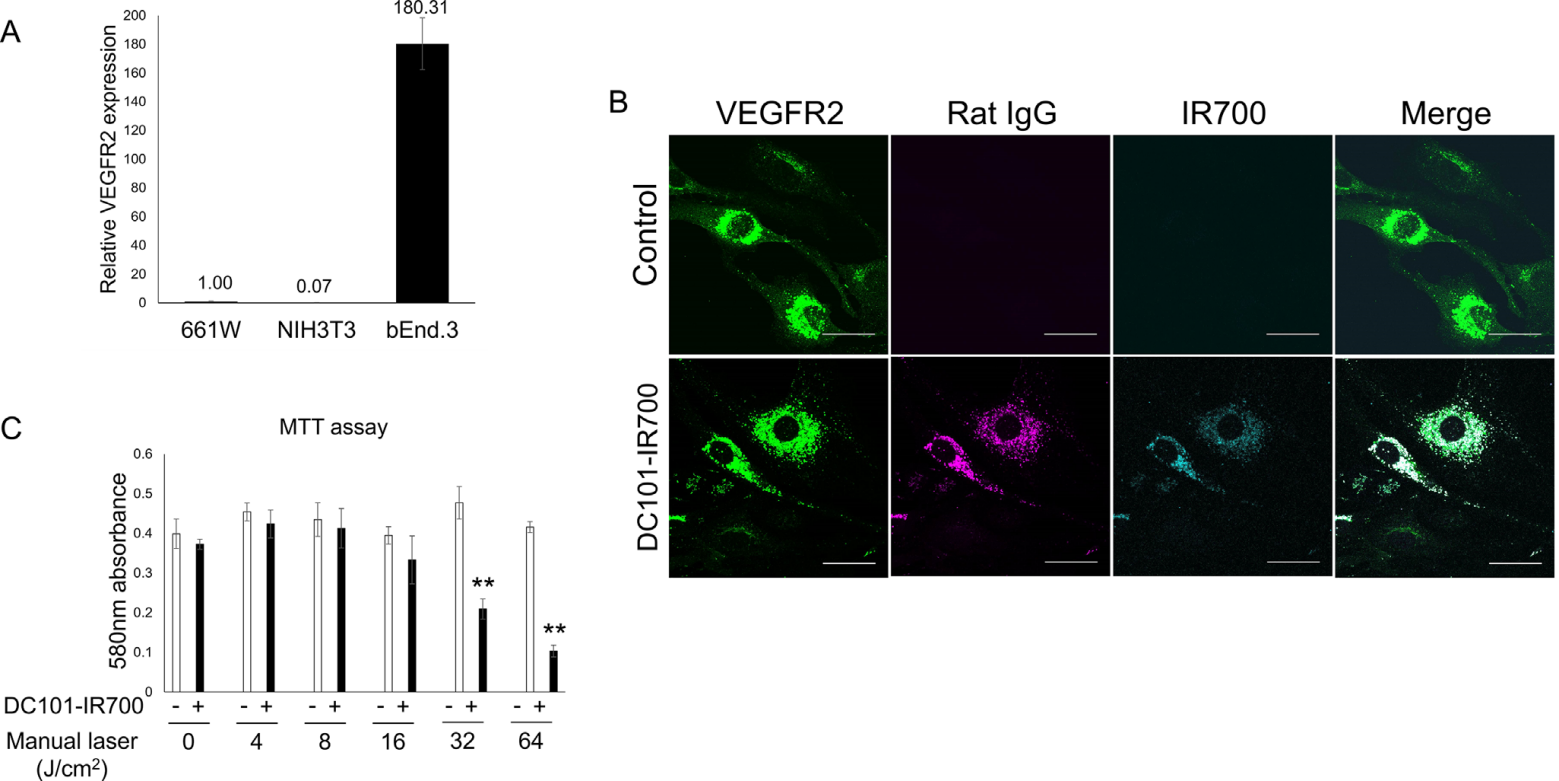
In vitro NIR-PIT in VEGFR2-expressing cells. (**A**) VEGFR2 mRNA is highly expressed in b.End3 cells compared with other cell lines. Data are presented as the means ± SEM (*n* = 3; **P < 0.01, one-way ANOVA with Tukey post hoc test). (**B**) Immunofluorescent staining for VEGFR2 and DC101-IR700 in b.End3 cells. (**C**) MTT assay data showing NIR irradiation-dependent cellular damage following NIR-PIT. Data are presented as the means ± SEM (*n* = 4; *P < 0.05, **P < 0.01, Student's *t* test). *Scale bar*, 30 µm.

We initially assessed the effectiveness of PIT in vitro, targeting VEGFR2-expressing endothelial cells. To quantify VEGFR2 expression in cultured cells, we conducted real-time PCR and immunohistochemistry using selected cell lines. VEGFR2 mRNA expression was significantly higher in b.End3 cells compared with other cell types, indicating this cell line is suitable for subsequent in vitro analyses (Fig. 1A). To confirm the intracellular localization of DC101, IR700-conjugated DC101 (DC101-IR700) was added to the culture medium, followed by immunostaining after 24 hours. IR700 signal colocalized with both the VEGFR2 antibody and Rat IgG-Alexa555 (Fig. 1B). No signals for rat IgG or IR700 were detected in cells that did not receive DC101-IR700 treatment (Fig. 1B). This finding suggests that IR700 successfully binds to endogenous VEGFR2 in b.End3 cells. Next, we evaluated the cytotoxic effects of NIR PIT on VEGFR2-expressing b.End3 cells using the MTT assay. Cells treated with DC101-IR700, an antibody–photosensitizer conjugate, were irradiated with NIR manual laser. PIT induced cell death in a NIR intensity-dependent manner (Fig. 1C). These findings confirmed that PIT effectively induces cytotoxicity in VEGFR2-expressing cells, supporting its potential as a therapeutic modality for targeting pathological neovascularization in AMD.

### Localization of DC101 Fluorophore Complex in CNV Lesions

**Figure 2.**
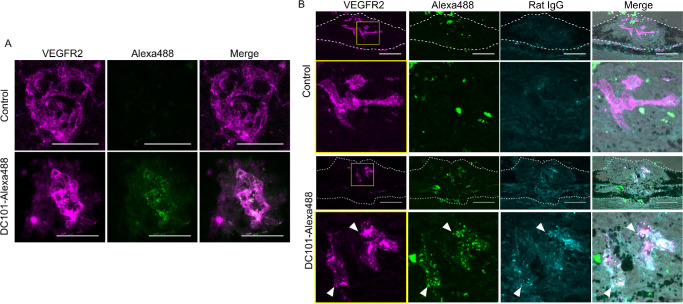
Localization of DC101 fluorophore complex in CNV lesions. (**A**) Flat-mount immunofluorescence imaging of CNV lesions in laser-induced CNV mouse retinas. In control mice, VEGFR2-positive neovascular structures were detected, but no Alexa488 signal was observed. In contrast, in DC101-Alexa488-treated mice, Alexa488 signals were observed within VEGFR2-positive neovascular regions in CNV lesions. (**B**) Tissue section analysis of CNV lesion. Although VEGFR2 expression is observed, no Alexa488 localization was observed in the CNV of the control group. In the DC101-Alexa488–treated group, DC101 indicated by Alexa488 and rat IgG signals, was localized in the CNV, and colocalized with VEGFR2. The bottom rows are an enlarged view of the *yellow boxed areas*. Punctate Alexa488 signals that colocalize with both VEGFR2 and Rat IgG (*arrowheads*) were observed in the DC101-Alexa488–treated group. The CNV lesions are demarcated by dotted lines. *Scale bar*, 50 µm.

To determine whether the antibody–fluorophore complex specifically localizes to CNV lesions in vivo , we conducted choroidal flat-mount immunofluorescence imaging in a mouse laser-induced CNV model. In the control group, VEGFR2-positive neovascular structures were detected, but no Alexa488 signal was observed. In contrast, in mice treated with DC101-Alexa488, Alexa488 signals were observed within VEGFR2-positive neovascular regions in CNV lesions (Fig. 2A). These results support the selective distribution of the DC101–fluorophore complex to VEGFR2-expressing regions within CNV lesions.

To further elucidate the precise localization of DC101 within CNV lesions, tissue sections were prepared and co-immunostained with an anti-VEGFR2 and anti-rat IgG antibody to detect endogenous VEGFR2 and rat-origin DC101. In the control group, Alexa488 fluorescence was observed in CNV lesions. However, these signals did not colocalize with either endogenous VEGFR2 or Rat IgG and were therefore considered nonspecific. In the DC101-Alexa488-treated group, similar nonspecific Alexa488 signals were also observed. In addition to these, punctate Alexa488 signals clearly colocalized with both endogenous VEGFR2 and Rat IgG (Fig. 2B), indicating that the antibody–fluorophore complex specifically binds to VEGFR2-expressing cells in CNV lesions. These results suggest that DC101-based PIT has the potential to selectively target neovascular lesions in CNV with high specificity.

### Reduction of CNV Volume by PIT

**Figure 3.**
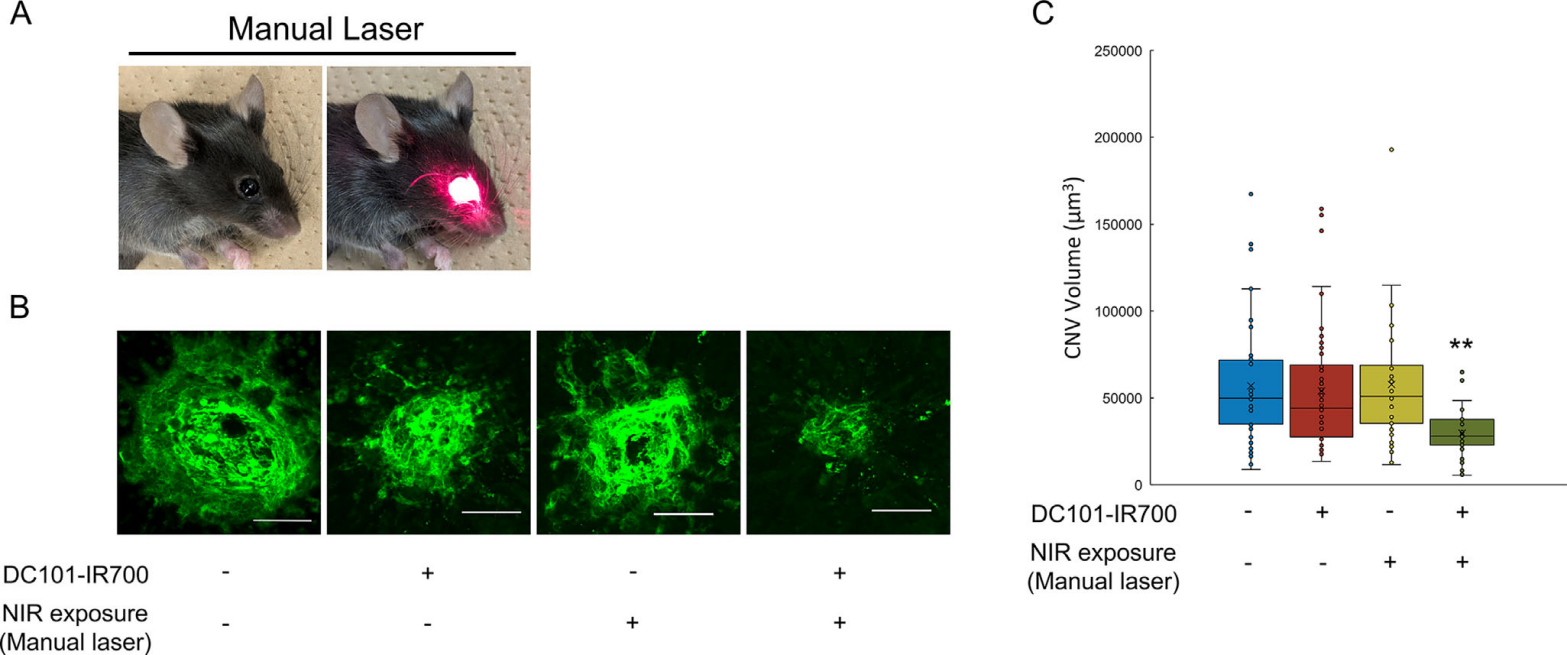
Reduction of CNV volumes by PIT using 690 nm NIR manual laser. (**A**) Manual laser irradiation method. (**B**) Images of isolectin B4 staining. (**C**) Quantification of CNV volumes 7 days after PIT. Significant CNV volume reduction was observed in the antibody-treated and NIR-irradiated group compared with controls. No CNV volume reduction was observed in the antibody-only or NIR-only groups. Data are presented as the means ± SEM (*n* = 38, 53, 35, 39; **P < 0.01, one-way ANOVA with Tukey post hoc test). *Scale bar*, 50 µm.

To evaluate the therapeutic efficacy of PIT, we examined its effects on CNV volume reduction in the laser-induced CNV mouse model. Mice were treated with DC101-IR700 and subjected to NIR manual laser irradiation to activate the PIT process (Fig. 3A). PIT resulted in a significant reduction in CNV volumes compared with the control group (Figs. 3B, 3C). No significant decrease in CNV volume was observed in groups that received only antibody treatment or NIR irradiation alone. These findings indicate that the combination of antibody administration and NIR irradiation is essential to achieving CNV volume reduction.

### Evaluation of Slit Lamp Laser In Vitro

**Figure 4.**
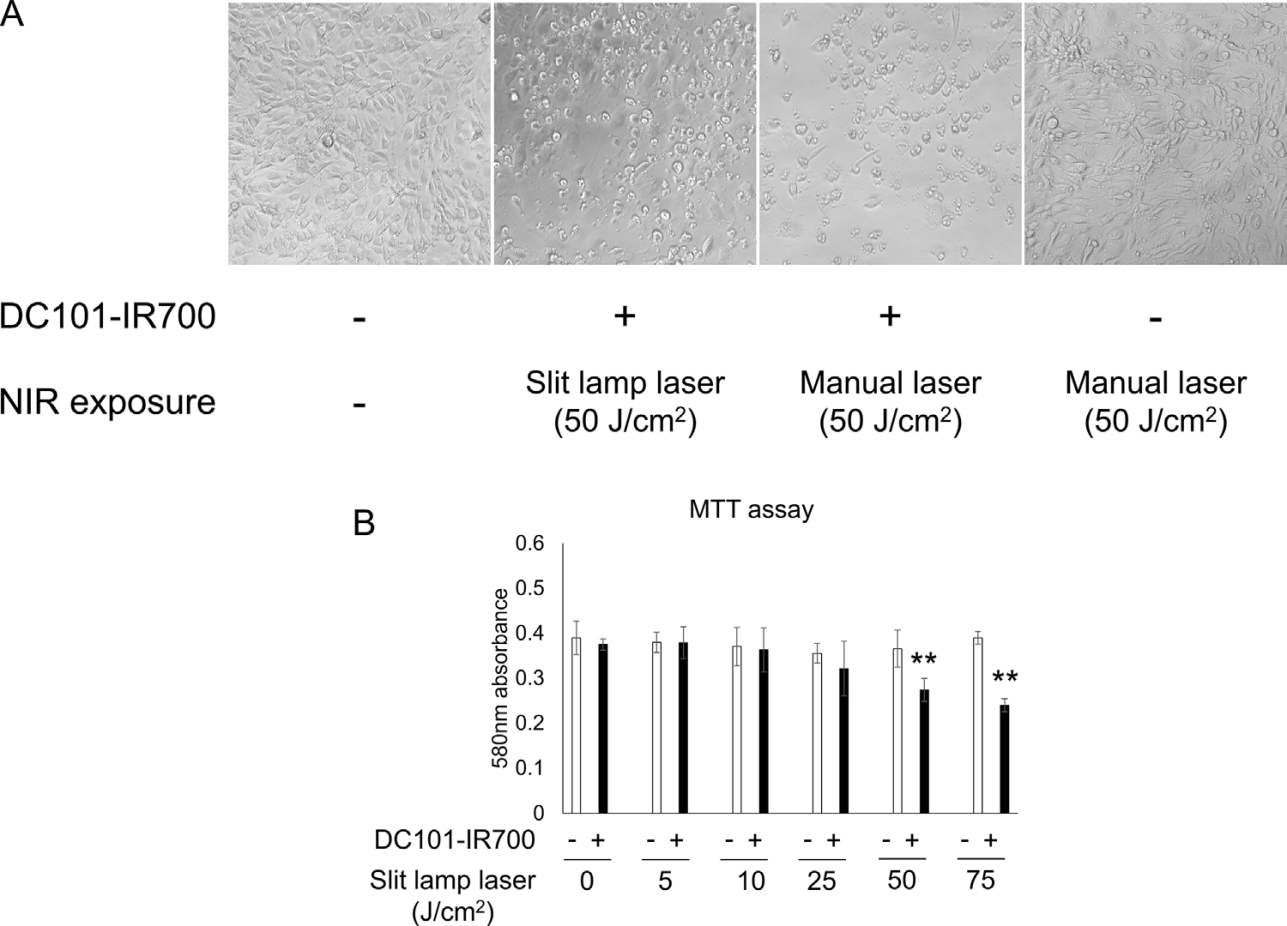
Induction of cell death by in vitro directional PIT. (**A**) Bright-field images 24 hours after PIT. Cell shrinkage was observed following NIR manual laser or slit lamp laser irradiation, but not with laser irradiation alone. (**B**) MTT assay data showing NIR irradiation-dependent cellular damage following directional PIT. Data are presented as the means ± SEM (*n* = 4; **P < 0.01, Student's *t* test).

Given that photodynamic therapy (PDT), a readily available treatment for AMD, uses a 690-nm slit lamp laser, we explored the laser's adaptability for PIT in clinical practice. We first evaluated its efficacy in vitro by irradiating DC101-IR700-treated cells with either the manual laser (PIT) or the slit lamp laser (directional PIT). Directional PIT induced cell shrinkage, which indicates cytotoxicity, thus demonstrating the potentials of this combination approach in inducing cell death (Fig. 4A). This change was not observed under the conditions where laser irradiation was performed without DC101-IR700 administration (Fig. 4A) or when the antibody was administered without laser irradiation (photo not shown). MTT assay results at 24 hours after directional PIT showed increased cell death in a slit lamp laser intensity-dependent manner (Fig. 4B). These findings suggest that the slit lamp laser, which is already established in clinical practice, can be used to achieve targeted cell death in neovascular tissues when combined with PIT.

### Reduction of CNV Volume by Directional PIT

**Figure 5.**
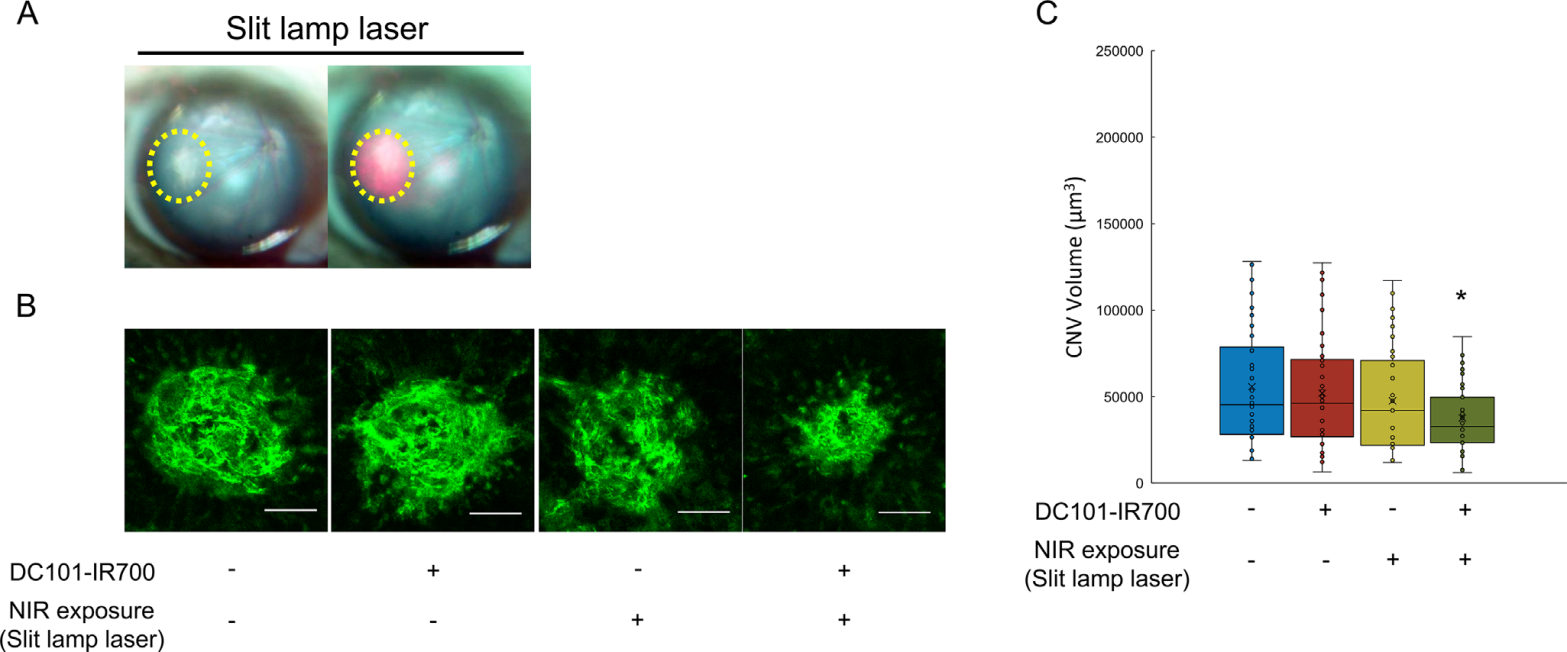
Reduction of CNV volumes by PIT using slit lamp laser. (**A**) Fundus during slit lamp laser irradiation. The CNV lesion is indicated by a *yellow dotted line*. (**B**) Images of isolectin B4 staining. (**C**) Quantification of CNV volumes. Significant CNV volume reduction was observed in the antibody-treated and slit lamp laser-irradiated group compared with controls. No CNV volume reduction was observed in the antibody-only or slit lamp laser-only groups. Data are presented as the means ± SEM (*n* = 37, 39, 37, 35; *P < 0.05, one-way ANOVA with Tukey post hoc test). *Scale bar*, 50 µm.

After confirmation of directional PIT's in vitro efficacy, we investigated its therapeutic potentials in vivo using the laser-induced CNV model mouse. The use of a slit lamp laser enables targeted irradiation of the CNV lesion (Fig. 5A). Similar to the results observed with PIT, directional PIT led to a significant reduction in CNV volumes (Figs. 5B, 5C). Mice that received both antibody treatment and slit lamp laser irradiation exhibited a marked decrease in CNV volume compared with controls. No significant decrease was observed in mice treated with either antibody or slit lamp laser alone. These results demonstrate that directional PIT effectively targets and reduces CNV volume in vivo, suggesting its potential as a novel therapeutic strategy for AMD with minimal effect on normal tissues.

### Induction of Cell Death in CNV Lesions by Directional PIT

**Figure 6.**
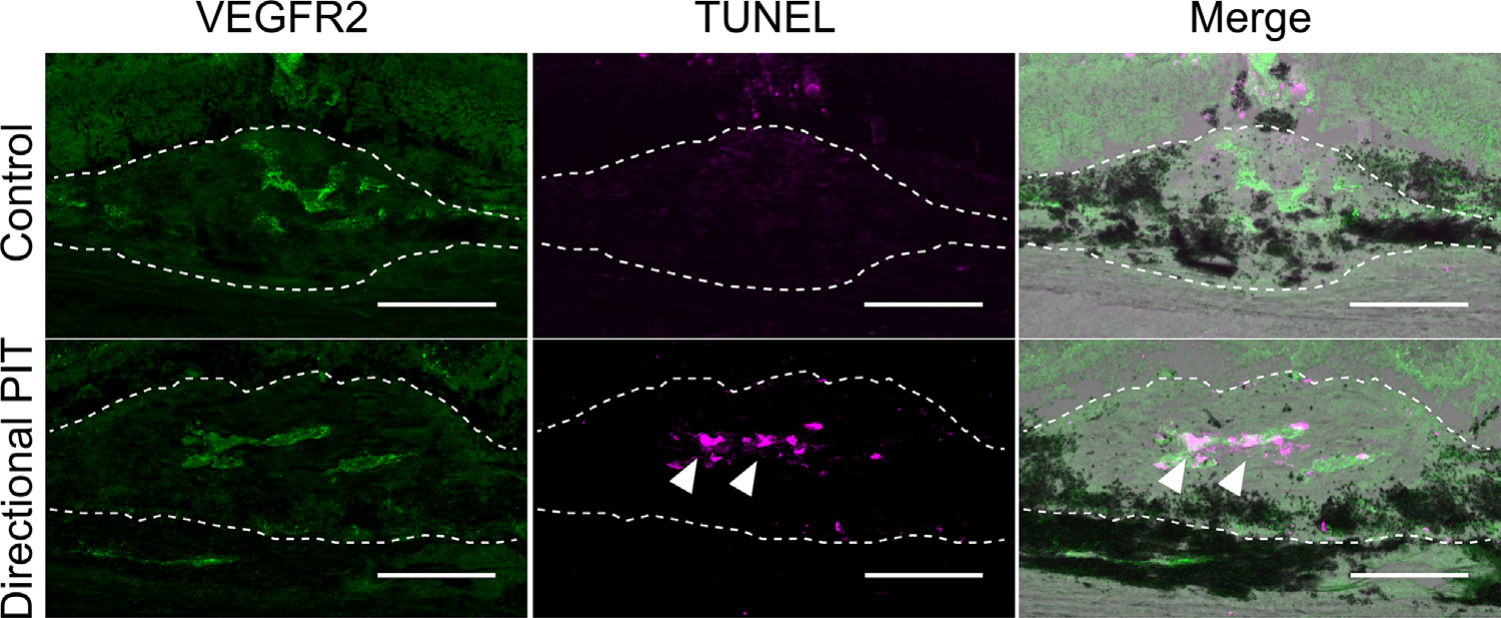
Directional PIT-induced cell death in CNV lesions 24 hours post treatment. In the control group, VEGFR2 expression was observed in the CNV, but no TUNEL-positive cells were detected. In the directional PIT group, TUNEL-positive cells were observed in VEGFR2-positive cells within the CNV lesions (*arrowhead*). The CNV lesions are demarcated by dotted lines. *Scale bar*, 50 µm.

To further validate the therapeutic efficacy of directional PIT, we assessed cell death within CNV lesions after treatment. At 24 hours after directional PIT, TUNEL staining was performed to detect cell death within CNV lesions. In the control group, VEGFR2 expression was observed in CNV lesions, but no TUNEL-positive cells were detected. In contrast, in the directional PIT-treated group, numerous TUNEL-positive cells were detected within VEGFR2-positive regions of CNV lesions (Fig. 6), indicating that directional PIT induced cell death in VEGFR2-expressing cells within neovascular tissue. These results support the potential of directional PIT as a viable therapeutic approach for targeting and inducing apoptosis in abnormal neovascularization, such as that observed in AMD. The induction of apoptosis specifically in VEGFR2-positive cells highlights the precision of this treatment modality.

## Discussion

Current treatment options for neovascular AMD such as anti-VEGF therapy, although clinically proven in many cases, have several limitations, including the need for frequent intravitreal injections, incomplete response in some patients, and potential adverse effects. In this study, we aimed to apply PIT to the treatment of CNV and to develop a novel treatment for neovascular AMD.

First, we showed the effectiveness of PIT in vitro by demonstrating cytotoxicity in VEGFR2-expressing cells. Next, we identified the localization of DC101 fluorophore complex in CNV lesions in vivo using mouse laser-induced CNV model. Finally, we demonstrated the decrease in CNV volume by this DC101-IR700 PIT. Additionally, we also showed the same CNV reduction using 690-nm slit lamp laser, which has been used widely for PDT targeting AMD.

Our study demonstrated that PIT effectively induced cell death in VEGFR2-positive cells within CNV lesions, resulting in a significant decrease in CNV volume. This finding suggests that the therapeutic effect of PIT is closely linked to its ability to selectively target and eliminate pathological neovascular cells expressing VEGFR2. Previous studies have explored alternative methods for inducing cell death in CNV lesions, such as the use of plasma-activated medium and CCR3 inhibition, both of which have been reported to decrease CNV volume by inducing apoptosis in neovascular tissues.^25^^,^^26^ Although these approaches offer additional therapeutic avenues, PIT stands out owing to its unique combination of antibody specificity and light-activated precision, which minimizes off-target effects and enhances safety. The confinement of PIT-induced cell death to CNV lesions, despite VEGFR2 being present in normal vasculature, highlights the relevance of VEGFR2 expression levels in pathological angiogenesis. Multiple studies have reported that VEGFR2 expression is significantly greater in neovascular endothelial cells compared with normal vessels, emphasizing its key role in driving pathological vascular growth.^27^ VEGFR2 is expressed predominantly in endothelial cells and is upregulated notably in conditions such as tumor vasculature, where it facilitates angiogenesis and disease progression.^28^ These findings suggest that the elevated VEGFR2 expression in neovascular endothelial cells makes them selectively vulnerable to PIT. This finding supports the hypothesis that VEGFR2 expression thresholds play a pivotal role in the therapeutic specificity of PIT, reinforcing its potential as a targeted therapy for pathological angiogenesis.

The ability of PIT to achieve significant therapeutic effects in a single treatment session represents a notable advancement over conventional anti-VEGF therapies, which often require frequent administrations. Similarly, previous studies have demonstrated the therapeutic effects of DC101, but these modalities also required repeated administrations to maintain efficacy.^19^^,^^29^ In this regard, the most unique advantage of PIT is attributable to its targeted elimination of VEGFR2-expressing cells, which play a pivotal role in pathological angiogenesis. By eradicating these key contributors to neovascular growth, PIT disrupts the angiogenic progression at its source. This process contrasts with traditional anti-VEGF approaches, which inhibit signaling without directly targeting the cells responsible for angiogenesis, necessitating ongoing treatments to sustain efficacy.

There are concerns that PIT for AMD may cause relatively severe complications such as bleeding, RPE tear, and late-period chorioretinal atrophy, as these are known complications of PDT for AMD.^30^^–^^32^ PDT, the first officially approved treatment of neovascular AMD, similarly uses specific light-sensitive drugs (e.g., porphyrin derivatives) and irradiates abnormal blood vessels with laser. In this treatment, verteporfin accumulates in abnormal blood vessels and is activated by 689-nm laser irradiation, which regresses the blood vessels thus suppresses bleeding and exudative changes. PDT is now often used as a combination therapy because its effectiveness is limited compared with anti-VEGF therapy. PIT is inherently different from PDT owing to its molecular targeting of VEGFR2. In addition, as described elsewhere in this article, the mechanism of action in CNV regression is also distinctly different from that of PDT. Therefore, the effects of PIT seem to be limited to only CNV and should spare surrounding normal tissues from damage.

In conclusion, we have demonstrated that PIT targeting VEGFR2 could be a novel treatment for neovascular AMD. By directly targeting VEGFR2-positive cells, PIT and directional PIT offer a more sustained and potentially effective approach to pathological angiogenesis. These therapies could not only alleviate the treatment burden for patients, but also provide an alternative for those who show limited responses to current anti-VEGF treatments. However, further research is needed to comprehensively evaluate the efficacy, safety, and feasibility of PIT in comparison with existing therapies, particularly regarding long-term outcomes and applicability across diverse patient populations.
